# The rise and fall of excess winter mortality in New Zealand from 1876 to 2020

**DOI:** 10.1007/s00484-023-02573-6

**Published:** 2023-11-27

**Authors:** Lucy Telfar-Barnard, Michael G. Baker, Nick Wilson, Philippa Howden-Chapman

**Affiliations:** Department of Public Health, University of Otago, Wellington, New Zealand

**Keywords:** Excess winter mortality, Trends, Seasonality, New Zealand, Living standards, 1918 pandemic influenza, Avoidable mortality

## Abstract

Excess winter mortality (EWM) has been used as a measure of how well populations and policy moderate the health effects of cold weather. We aimed to investigate long-term changes in the EWM of Aotearoa New Zealand (NZ), and potential drivers of change, and to test for structural breaks in trends. We calculated NZ EWM indices from 1876 (4,698 deaths) to 2020 (33,310 deaths), total and by age-group and sex, comparing deaths from June to September (the coldest months) to deaths from February to May and October to January. The mean age and sex-standardised EWM Index (EWMI) for the full study period, excluding 1918, was 1.22. However, mean EWMI increased from 1.20 for 1886 to 1917, to 1.34 for the 1920s, then reduced over time to 1.14 in the 2010s, with excess winter deaths averaging 4.5% of annual deaths (1,450 deaths per year) in the 2010s, compared to 7.9% in the 1920s. Children under 5 years transitioned from a summer to winter excess between 1886 and 1911. Otherwise, the EWMI age-distribution was J-shaped in all time periods. Structural break testing showed the 1918 influenza pandemic strain had a significant impact on trends in winter and non-winter mortality and winter excess for subsequent decades. It was not possible to attribute the post-1918 reduction in EWM to any single factor among improved living standards, reduced severe respiratory infections, or climate change.

**Supplementary Information:**

The online version contains supplementary material available at 10.1007/s00484-023-02573-6.

## Background

Excess winter mortality (EWM) describes the phenomenon of higher mortality rates in winter than in summer or outside winter. EWM is observed worldwide and tends to be highest in temperate climates. It is often used as a crude indicator of people’s vulnerability to cold, or alternatively as a measure of how well a society tempers the effects of cold, in particular through the quality of housing. However, there is still debate about how much of EWM is cold-related and how much is due to other seasonal factors (Staddon et al. 2014).

EWM is most commonly measured by comparing the mortality rate for the four coldest months to the mortality rate over the combined previous and subsequent 4-month periods. This ratio is the EWM Index (EWMI) (Curwen 1991), also known as Curwen’s Index. In the Southern Hemisphere, raw EWMI is measured as:

$$\frac{(Deaths\;June\;to\;September)\;\div\;122}{\left(Deaths\;February\;to\;May\;+\;Deaths\;October\;to\;January\right)\;\div\;243\;\lbrack244\;\mathrm{in}\;\mathrm{leap}\;\mathrm{years}\rbrack}$$

There are justified criticisms of EWM as a measure of temperature-related mortality (Hajat and Gasparrini 2016). Where possible, the cold temperature-mortality relationship is better measured using time-series models (Gasparrini et al. 2015). Such models remove seasonal trends from data and so do not capture all winter deaths. However, they are more sophisticated than EWM ratios, as they allow greater control for correlated exposures such as air quality or humidity; do not rely on arbitrary dates to distinguish winter from non-winter; and separate the effects of summer from the effects of winter (Hajat and Gasparrini 2016). Nevertheless, EWM remains a useful if crude measure, being straightforward to calculate, unaffected by the availability or quality of temperature data, and simple both to convey and to understand. It has also been suggested as useful for comparing severity of historical influenza epidemics (Jones and Ponomarenko 2022).

EWMI magnitude is highly age-stratified, with the highest levels appearing in the very young and the elderly. Differences by sex vary by study: women are usually (Wilkinson et al. 2004; Telfar Barnard 2010; Maheswaran et al. 2004; Davie et al. 2007), but not always (Hales et al. 2012), found to have higher EWMIs than men. Seasonal and pandemic influenza is a large contributor to the EWM burden, contributing not only through respiratory deaths, but also through cardiovascular disease mortality (Jackson et al. 2014; Tillett et al. 1983). Dementia and Alzheimer’s disease have also been identified as contributing to the EWM burden (Excess winter mortality in England and Wales 2020).

Although seasonality in illness has been recognised since at least Hippocrates (Hippocrates 400 B.C.E.), there are few longitudinal studies of more than 30 years of either EWM or the temperature-mortality curve. Those few studies find people’s risk of death from the cold has decreased over time and ascribe this decrease to improvements in social, environmental, behavioural and/or health-care factors (Carson et al. 2006; Chau and Woo 2015; Ekamper et al. 2009; Jones and Ponomarenko 2022; Lerchl 1998; Marcuzzi and Tasso 1992; McDowall 1981), or population aging (de Schrijver et al. 2022), with Hare et al. observing that the effect of any individual factor may not remain constant (1981).

Aotearoa New Zealand (NZ) is a high-income country where EWM has been well-described for the period 1980 to 2000 (Davie et al. 2007), but longer-term historical trends have not been examined. In this study, we aimed to examine changes in EWM and the seasonal distribution of mortality in NZ over the 145-year period from 1876 to 2020, and consider potential contributions to those changes over time.

## Methods

**Fig. 1:**
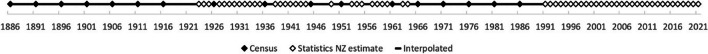
Population data sources by year 1886 to 2021. Census years are five yearly from 1886 to 1892 to 1916, then 1926, 1936, 1945,and 1951, then five yearly from 1961 to 1986. Statistics NZ estimates were available for 1923 to 1925, 1927 to 1935, 1938 to 1944, 1949, 1953 to 1960 and from 1991 to 2021 inclusive. Remaining years were interpolated

We obtained mortality data from 1 January 1876 to 31 January 2021 inclusive, from the NZ Department of Internal Affairs (1 January 1876 to 31 December 1999) and the Ministry of Health (1 January 2000 to 31 January 2021). Age- and sex-stratified population data from 1886 to 2021 were established by combining data from a range of Statistics NZ sources: NZ yearbooks, both physical and online, and population estimates and Census counts from Statistics NZ (nz.stat 2021), and then using linear interpolation by 5-year age group and sex to estimate data for the remaining years. Available population data were not disaggregated by age and sex before 1886, and we did not consider it safe to extrapolate these earlier years as the population was changing rapidly in this colonial period. Population data sources by year are shown in Fig. 1.

Mortality data were manually cleaned to remove deaths that had occurred overseas. For pre-2000 data, this cleaning involved text matches, text searches and sorting by location of death to identify overseas locations. For post-2000 data, overseas deaths had a “9999” place of death location code.

We first counted total deaths and absolute EWM (winter deaths − (non-winter deaths × winter days/non-winter days)) per year from 1876 to 2020. We then calculated 5-year age group–specific EWMIs for 36-year periods starting February 1876 to January 1912 and ending with February 1984 to January 2020. For November and December 1918 in the 1912 to 1947 period, we used dummy variables repeating November and December 1917 deaths, to remove the substantial mortality effect of the 1918 influenza pandemic in NZ. We also calculated 5-year age group- and sex-specific EWMIs for the two halves of the study period, February 1876 to January 1948 and February 1948 to January 2020, and used robust Poisson regression to estimate relative rate ratios of female to male EWMIs by 5-year age group for each half of the study period.

Next, for the period 1886 to 2020, we calculated total mortality rates, age- and sex-standardised to Census year 2018, for 4:8 month winter (June to September) and non-winter (February to May, October to January) periods, and the age-standardised EWMI for these years, total and by 5-year age groups to 80 years and over.

While we included 2020 for completeness in year-on-year graphs, 2020 was excluded from group and time-trend analyses, to prevent any skew from the heavily suppressive effect of Covid-19 pandemic control measures on winter illness mortality rates (NZ experienced negative total excess mortality in the 2020 and 2021 years (Knutson et al. 2023; Ledesma et al. 2023)). We did not exclude the years affected by poliomyelitis epidemic control measures in the summers of 1924–1925, 1936–1937 or 1947–1948 (Ross 1994), as there was no visible effect of these measures on total mortality or EWMs for those years, compared to years either side.

After removing time-trends by subtracting 10-year rolling averages, we used *t*-tests to test for differences in variance between winter and non-winter mortality rates over the full time period, and before and after 1947.

### Structural breaks

We used Ditzen et al’s “xtbreak” command (Ditzen et al. 2021), with 5% trimming, to test for multiple (up to 14) possible break points, and for a structural break in 1997 (the introduction of free influenza vaccination for people aged 65 years and over). Structural break testing was carried out with data for all ages and with data for people aged 65 years and over.

### Spatial trends

Place of death was included in available data for some deaths from 1876 to 2014, as a free-text field from 1876 to 1987 and a “domicile code” from 1988 to 2014. Where the free-text field could be matched to a unique suburb or city, we assigned them to a Territorial Authority area. Domicile codes were also matched to Territorial Authority areas using Ministry of Health matching files. Deaths were then assigned to either North Island or South Island. Age-disaggregated population data by region or island were not available for earlier decades; however, we calculated annual EWMIs for North and South Islands, adjusted to each island’s age distribution of deaths in 2010, to assess how population drift might have impacted total EWMIs over time.

All statistical analysis was carried out in Stata/MP 17.0.

The University of Otago Ethics Committee granted ethics approval for this research (HD19/073).

## Results

**Fig. 2:**
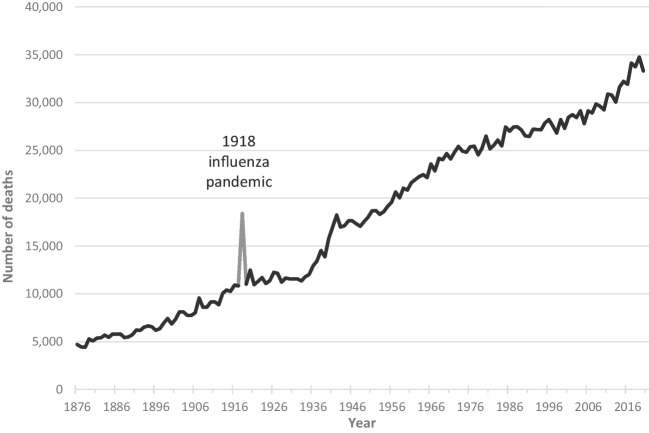
Number of deaths in New Zealand per year from 1876 to 2020. Figure shows the number of deaths rising from just under 5000 in 1876 to 35,000 in 2020. Numbers vary from year to year, but the rise is generally steady except for a marked spike of 18,397 in 1918, compared to around 11,000 in 1917 and 1920. Numbers increased little between 1920 and 1935 but rose more steeply from 1935 to 1942

Total deaths per year increased linearly by 211.0 deaths per year, from 4,698 in 1876 to 34,752 in 2019 (Fig. 2). Deaths per year flattened after the 1918 influenza pandemic, but also rose less steeply between the 1980s and the 2000s.

**Table 1:** Mean excess winter mortality indices and absolute winter mortality excess and absolute excess as a percentage of total deaths, by decade, and potentially relevant historic events, 1880s to 2010s

Decade	Mean EWMI (age- and sex-standardised)	Mean absolute EWM^a^	% of total deaths	Notable events in New Zealand society
1880s		− 30	− 0.1	First successful shipment of frozen meat to Britain in 1882 reflects a surge in economic growth for NZ. Long economic depression from the late 1870s through to the early 1890s. Sanitation in NZ towns and cities was poor, resulting in elevated infectious disease mortality, particularly enteric infections which are associated with summer.
1890s	1.19	150	2.3	Coldest decade in this 145-year period.
1900s	1.20	261	3.2	NZ population reached 1 million in 1908.
1910s	1.20	426	4.2	First World War (WW1) spanned 1914 to 1918 with high male mortality — albeit adjusted for in the NZ mortality data by excluding deaths overseas. The 1918 pandemic influenza strain may have continued to cause increased influenza deaths in several subsequent years
1920s	1.34	923	7.9	Increasing urbanisation and home ownership. Public health and social measures (PHSM) for polio prevention used in the non-winter period of 1924–1925.
1930s	1.25	762	6.1	The “Great Depression” starts following the 1929 Wall Street Stock Market Crash. NZ formed a coalition government to combat the depression in 1931. Social welfare initiatives were introduced. PHSM for polio prevention (non-winter 1936–1937) (Ross 1994).
1940s	1.23	1059	6.1	Second World War (WW2) spanned 1939 to 1945 with high male mortality — albeit adjusted for in the NZ mortality data. PHSM for polio prevention (non-winter 1947–1948). Steepest rise in temperatures occurred between 1940 and 1960 (Mullan et al. 2010 ).
1950s	1.29	1439	7.4	An influenza pandemic in 1957 increased mortality (Wilson et al. 2012).
1960s	1.25	1576	7.0	An influenza pandemic in 1968 increased mortality. Māori deaths combined with total deaths rather than collected separately after 31 December 1961 (The New Zealand official yearbook 1962 ).
1970s	1.22	1478	5.9	First Building Code for insulation in housing introduced in 1978.
1980s	1.20	1527	5.7	Economic reforms cause shocks to the economy and increase unemployment levels. Increases in Māori life expectancy stall (Woodward and Blakely 2015).
1990s	1.21	1687	6.2	Economic reforms continue — causing difficulties for some sectors of society. In 1996. there was a relatively severe influenza A(H3N2) epidemic. Influenza vaccine added to the immunisation schedule for adults aged 65 years and older (1997) and extended to those aged under 65 years with certain medical conditions (1999) with impact potentially seen in subsequent decades.
2000s	1.16	1423	4.9	More than half of NZ’s 1.6 m homes estimated to have no or substandard insulation.
2010s	1.14	1450	4.5	The warmest decade in this 145-year period. Government subsidy scheme retrofitted insulation in ~ 345,000 homes, but roughly a quarter of homes remained un- or under-insulated by Building Code standards at end of decade (Annual Report 2017/2018; Annual Report 18/19, 2019; Annual Report: 1 July 2019–30 June 2020, 2020).
2020 only	1.02	225	0.7	Covid-19 (SARS-CoV-2) pandemic control measures result in a near total elimination of circulating influenza, RSV and other serious respiratory infections in 2020 (Huang et al. 2021) and an associated reduction in mortality rates and the EWMI.

^a^Absolute EWM = number of excess deaths

**Fig. 3:**
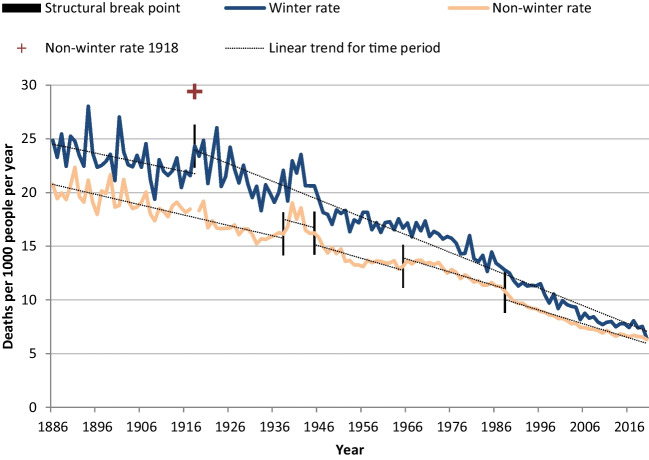
New Zealand winter and non-winter mortality rates 1886 to 2020, standardised by 5-year age-groups and sex. Figure shows a decline in mortality rates for both winter and non-winter. The winter line runs from around 25 deaths per 1000 people per year in 1886 to 7.25 in 2019, with a break point in 1918. The linear trend after 1918 is steeper than before 1918. For non-winter, rates drop from 21 deaths per 1000 people per year in 1886 to about 6 in 2020, with a spike of 29.4 in 1918, and break points at 1938, 1945, 1966 and 1988. The linear trends within break sections are roughly similar in angle, but with a higher constant between 1938 and 1945, and 1966 and 1988, than in the other three periods

While total deaths increased with the population, mortality rates declined in both winter and non-winter periods (Fig. 3). Year-to-year variation in mortality rates became less volatile over time but retained more volatility in winter than in non-winter, confirmed by *t*-testing (*p* < 0.05)

**Fig. 4:**
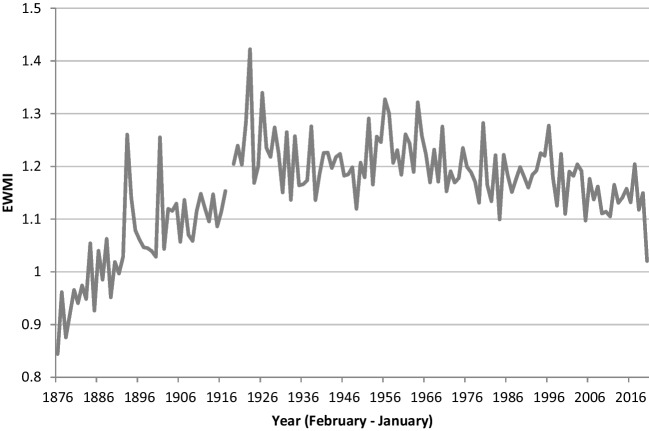
New Zealand unadjusted EWMIs for 1876 to 2020 (not showing the 1918 pandemic-related EWMI of 0.60). Figure shows high year to year variability in EWMIs. Overall EWMIs rise from around 0.9 to around 1.15 between 1876 and 1918, then increase to about 1.28 in the early 1920s, dropping back below 1.2 in the mid-1940s, rising again in the 1950s, and then declining over the remaining years. The highest point is 1.42 in 1923. 2020 is markedly lower than the previous trend

**Fig. 5:**
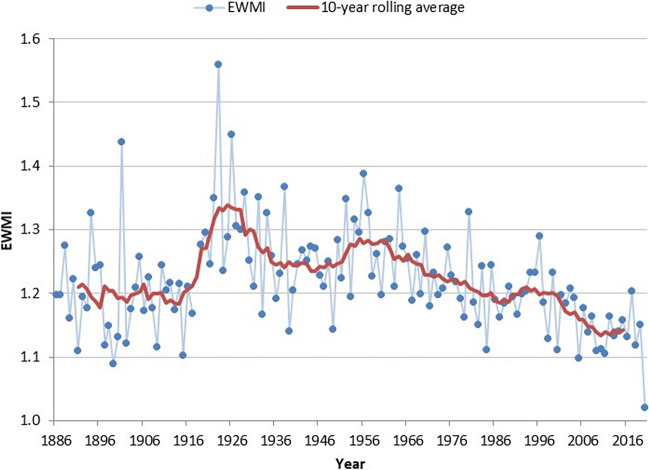
New Zealand annual EWMIs 1886 to 2020, standardised by 5-year age groups and sex (not showing the standardised 1918 pandemic-related EWMI of 0.82), with 10-year rolling average. Figure shows a similar pattern to Figure 4, except EWMIs no longer appear to increase between 1886 and 1918, varying around 1.2, then increasing to above 1.3 in the early 1920s. 2020 is now the lowest point on the chart

Unadjusted EWMIs for the period 1876 to 2019 showed a steady increase over the period 1876 to 1918, then a step-change upwards following the 1918 influenza pandemic (Fig. 4). Raw excess winter deaths peaked at 7.9% of total deaths in the 1920s and 7.4% in the 1950s, and then declined to 4.5% of total deaths in the 2010s (Table 1). The 2020 EWMI of 1.02 was the lowest since 1891. Age- and sex-standardised EWMIs averaged 1.22 from 1886 to 2019. EWMIs from 1886 to 1918 (average: 1.20) were volatile but with no meaningful gradient, then followed a similar path to unadjusted EWMIs, with a similarly gradual but not necessarily smooth or linear decrease in EWMIs after 1918, from an average of 1.34 in the 1920s to 1.14 in the 2010s (Fig. 5).

**Table 2:** Female EWMI to male EWMI relative rate ratios (RRR) by 5-year age group for periods 1876 to 1945 and 1946 to 2020

**RRR**	Age group																
Period	0–4	–-9	10–14	15–19	20–24	25–29	30–34	35–39	40–44	45–49	50–54	55–59	60–64	65–69	70–74	75–79	80+
1876 to 1945	0.98	**1.09** *	1.16**	1.07*	1.08*	1.12*	1.14**	1.07*	1.09*	0.95	1.03	0.97	0.99	1.01	1.02	1.01	0.99
1946 to 2020	1.02	1.22**	1.09	1.03	1.14**	1.10*	1.13**	1.01	0.99	1.03	1.01	1.01	1.03*	0.99	1.01	0.99	0.99

Figures in bold are statistically significant

*p < 0.05; **p < 0.001

**Fig. 6:**
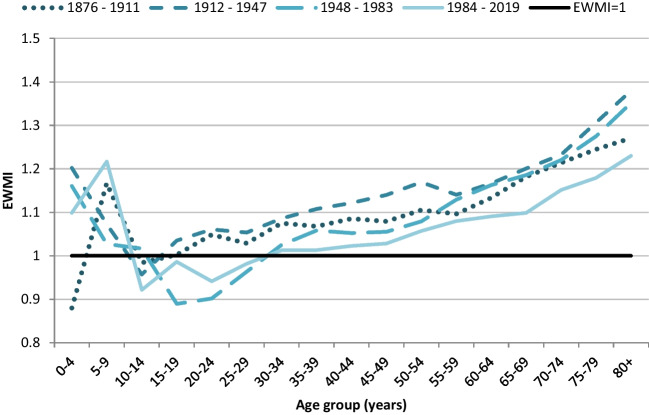
New Zealand EWMIs by 5-year age groups, in 36-year periods from 1876 to 2019. Figure shows J-shaped lines for 1912 to 1947 and 1948 to 1983, with high EWMIs in 0 to 4-year-olds, dropping to lowest EWMIs in 10 to 14-year-olds (or 15–19-year-olds for the 1948 to 1983 period), then increasing to highest EWMIs in 80+. In 1876 to 1911, the lowest EWMIs are in 0 to 4-year-olds, but high in 5- to 9-year-olds. The line for 1984 to 2019 is similar to the two previous periods except that EWMIs are higher in 5- to 9-year-olds than in 0- to 4-year-olds

In the 1876 to 1911 period, children aged under 5 years had a non-winter excess (EWMI: 0.88). Otherwise, EWM showed a J-shape across age groups, with lowest EWMIs in the 10- to 14- or 15- to 19-year age groups, and EWMIs increasing with subsequent age groups. All age groups from 30 to 34 years upwards showed a winter excess in all time periods (Fig. 6).

The gradient of increase in EWMI with increasing age was particularly steep in the 1948 to 1983 period, but was otherwise relatively similar, though starting from different low points.

**Fig. 7:**
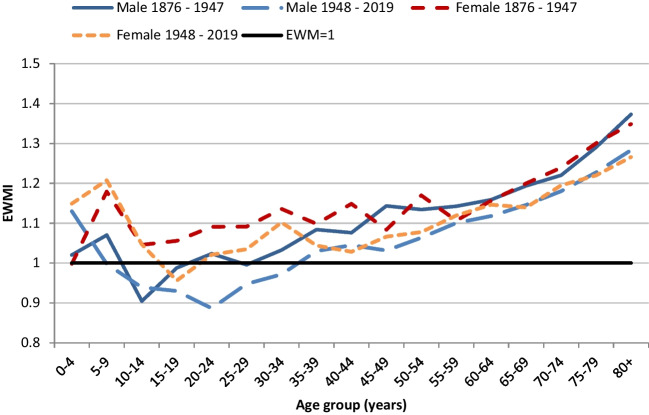
New Zealand EWMIs by 5-year age groups and sex, 1876 to 1847 and 1948 to 2019. Lines are similar to Figure 6. Differences between males and female EWMIs are shown in Table 2

Changes in EWMI between the first and second half of the study period were similar for males and females (Fig. 7). In the first half of the study period, females had significantly higher EWMIs than males in all age groups between 5 and 44 years, but male and female EWMIs were not meaningfully different under 5 years old, or for age groups 45 years and older. In the second half of the study period, female EWMIs were significantly higher than males in 5 to 9 year olds, and 20 to 29 year olds, but not otherwise meaningfully different. Relative rate ratios of female to male EWMIs by age group for each half of the study period are shown in Table 2.

**Table 3:** Structural breaks in linear trends for EWM, winter and non-winter mortality breaks

	EWMI	Winter mortality rates	Non-winter mortality rates
All ages			
Estimated structural breaks [break year(s)]	1^a^ [1918]	1^b^ [1918]	4^c^ [1938, 1945, 1965, 1988]
Breaks required to include year proximal to 1997 [proximal year]	10 [1996]	10 [1997]	7 [1999]
65 years and over			
Estimated structural breaks	1^d^ [1918]	4^e^ [1900, 1917, 1960, 1989]	4^f^ [1896, 1913, 1933, 1989]
Breaks required to include year proximal to 1997 [proximal year]	8 [1997]	3 [1996] 17 [1999]	9 [1999]

See Online Resource 1 for statistical details a–f

### Structural breaks

Testing found just a single structural break in EWMIs, in 1918 for all ages, and for ages 65 years and over. Testing did not reject the possibility of a break in 1997 for either age bracket; however, only when 10 breaks were estimated did 1996 appear as a potential break date for all ages, and only when eight breaks were estimated did 1997 appear as a potential break date for ages 65 years and over (Table 3).

For winter mortality rates, 1918 was again the single structural break year identified for all ages, but for ages 65 years and over, the test estimated breaks in 1900, 1917, 1960 and 1989. Again, while a 1997 structural break was not rejected as a hypothesis for either age bracket, 10 breaks were needed before a 1997 structural break point was included in estimates for all ages. For ages 65 years and over, estimates for three structural breaks included 1996 (with 1917 and 1980), but the four breaks had higher test statistics, and 17 breaks were needed before even 1999 was included.

For non-winter mortality rates, structural breaks were estimated in 1938, 1945, 1965 and 1988 for all ages and in 1896, 1913, 1933 and 1989 for ages 65 years and over. The hypothesis of a 1997 structural break point was not rejected, but 1999 was the closest break point estimated for all ages, included with seven breaks. For ages 65 years and over, nine breaks were needed before 1999 was included.

### Spatial trends

**Fig. 8:**
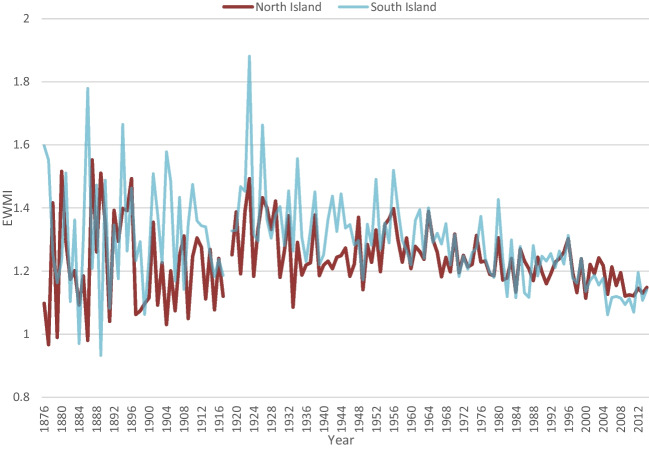
Age-adjusted North and South Island EWMIs 1876 to 2014. Lines are similar to description of Fig. 5, except that EWMIs for the North Island are generally lower than for the South Island until about the 1960s, at which point they become more similar. At the end of the study period, South Island EWMIs may be becoming lower than in the North Island. It is also worth noting that the reduction in EWMIs since 1918 appears steeper for the South Island than for the North Island

Match rates to Territorial Authority areas increased substantially over time, from 76% in the 1870s to 96% in the 2010s. Of those deaths that could be matched, the proportion of deaths in the North Island increased from 44% in the 1870s to 64% in the 1950s, up to 73% in the 2010s. Meanwhile, EWMIs in the North Island transitioned from 91% of South Island EWMIs in the 1870s and 1880s to 97% in the 1950s, to 103% in the 2000s and 2010s. Age-adjusted EWMIs by year for North and South Islands are shown in Fig. 8.

## Discussion

This study describes NZ’s EWM for the 145 years from 1886 to 2020, identifying important shifts in the seasonality of NZ’s deaths. Until the end of the First World War (WW1), EWMIs were volatile, but with no gradient over time. After the 1918 influenza pandemic, however, there was a sharp rise in EWM, and then a gradual decline over the rest of the study period, interrupted by an increase leading up to the 1957 influenza (H2N2) pandemic, and an additional peak with NZ’s 1996 influenza A(H3N2) epidemic. Population drift from north to south explains only a small proportion of the post-1918 decline in EWM. While young children had a summer excess in mortality in the early quarter of the study period, this excess transitioned to winter before WW1. Otherwise, the overall J-shaped age distribution was retained across the study period. Similarly, there was little change over time in sex differences: female EWMIs were generally higher than for male at younger ages, but converged in older adults.

While we can observe these distributions, attributing causation is more difficult. The study period 1876 to 2019 saw broad changes in NZ’s economic, social and health standards. Through the late nineteenth century and first half of the twentieth century, NZ living standards were among the highest in the world (Woodward and Blakely 2015). While NZ’s increase in living standards over the latter half of the twentieth century was not as great as in other high-income countries (Singleton 2008), it was still an increase. NZ introduced broad population vaccination programmes for a range of diseases from the 1940s on (Immunisation Handbook 2017, 2018). Of note, influenza vaccine was added to the immunisation schedule for older New Zealanders in 1997 and those with specific medical conditions in 1999 (Appendix 1: The history of immunisation in New Zealand 2020). NZ has had a social welfare system and an essentially fully publicly funded health care system since the 1930s, and large-scale state house construction occurred in the 1930s and 1940s, before and after the Second World War (WW2). However, overall, NZ housing has generally been relatively poorly insulated and inadequately heated by modern international benchmarks (Hindley 2020b, a). Mandatory standards for new builds have been improving slowly since the late 1970s, but until 2019 — and then only in private rentals (Residential Tenancies (Smoke Alarms and Insulation) Regulations 2016) — have not required existing housing to be retrofitted with insulation, despite growing evidence that retrofitting insulation in housing reduces hospitalisations and mortality (Fyfe et al. 2020; Howden-Chapman et al. 2007). Different government schemes to encourage improved insulation and/or more effective heating have been intermittently available since at least the 1970s (Thermal insulation in New Zealand homes 2020) and have been widely available since 2007, but even in 2015, only 33% of homes were assessed as insulated to current basic building code standards (White and Jones 2017). Climate disruption has also increased average outdoor temperatures in NZ over the study period, particularly post-WW2 (Indicators>Temperature 2020).

Statistical testing, across different testing methods, identified a structural break in EWMIs following the 1918 influenza pandemic. The 1918 pandemic also potentially shifted winter mortality rates, but did not appear to cause any significant change in non-winter rates. Other potential change dates identified by structural break testing do not neatly align with any specific policy interventions. While the introduction of vaccination in 1997 may have made some difference to EWM, or to winter or non-winter mortality rates, its impact was not substantial enough to stand out as a clear structural break point.

It seems reasonable to attribute the pre-WW1 shift from a summer to winter excess for children under 5 years to improved water and sewerage infrastructure in towns and cities preventing summer infectious disease mortality among infants (particularly from serious enteric diseases like typhoid, salmonellosis and cholera). Similar observations were made by Ekamper et al. for the Netherlands (2009), and in the epidemiologic transition that Carson et al. observed for London (2006).

In contrast, the post-WW1 structural break in EWMIs has not been observed elsewhere. England and Wales saw a gradual increase in seasonality ratios over the early twentieth century, peaking in 1930. Jones and Ponomarenko observed that the 1918 influenza pandemic in Denmark, the USA and Sweden occurred at a time of high baseline EWM, rather than introducing higher EWM (Jones and Ponomarenko 2022). Other long-term seasonality studies do not present their findings in a comparable way.

NZ’s overall decline in EWM after WW2 is, however, consistent with other studies’ findings of reductions in EWM or related measures over extended periods within that timeframe. EWM declines have been observed for England and Wales (McDowall 1981); Hong Kong (Chau and Woo 2015) Denmark, Sweden and the USA (Jones and Ponomarenko 2022); reductions in variation between cold and warm seasons have been observed in Italy (Marcuzzi and Tasso 1992), Germany (Lerchl 1998) and Australia (Hanigan et al. 2021), and a decrease in the cold temperature-mortality gradient has been observed in London (Carson et al. 2006; Carson et al. 2004) and Switzerland (de Schrijver et al. 2022).

Among the various improvements over time to health and living standards, excluding the clear effect of Covid-19 pandemic control measures in 2020, no single measure can be identified as having any substantial specific impact on NZ EWMIs. From 10-year rolling averages and structural break testing, we can see that neither the introduction of the social welfare system (1930s) nor state housing construction (1940s) prevented rises in EWM in the 1950s or late 1990s. Improvements in insulation requirements, in 1978, 1990, 2008 and 2017, may have contributed to reduction in EWMI over that period, but as they have applied only to new builds (or, from 2017, to properties with new rental agreements), they have affected only a very gradually increasing proportion of the total housing stock, meaning any effects on EWM would also be gradual rather than showing any break in full population effects visible or testable from the regulatory date. However, as national insulation levels remain substantially below those of other countries in comparable temperate climate zones, it is also possible that insulation levels may not yet be sufficiently improved to bring measurable reductions in EWM. Alternative research methods using individual exposure data, and a longer intervention period, would be needed to examine whether insulation subsidy programmes or the 2018 introduction of a home-heating subsidy (a “Winter Energy Payment”) are associated with an EWM reduction, as observed in the UK (Iparraguirre 2014; Viggers H, Ingham T, Chapman R, Crane J, Currie A, Davies C, Keall M, Pierse N (2023) RCT: Winter heating vouchers did not reduce COPD exacerbations, but may reduce mortality. In review.).

Climate change–induced warmer winters could also have contributed to the EWM reduction. Hanigan et al. have observed a reduction in Australia’s summer to winter mortality ratio between 1968 and 2018, which they attribute to summer temperatures having increased over this period (Hanigan et al. 2021). We observe that NZ EWM has reduced over the period during which the climate has been warming, but recognise that correlation is not causation. Research examining daily temperature-mortality relationships for the periods and locations where such temperature data are available is recommended, but beyond the scope of this study.

A further pathway that could potentially explain the steady decline in EWM over the last century is a reduction in the contribution of infectious respiratory diseases as a cause of death. This hypothesis is supported by the observation that severe infectious respiratory diseases have an association with age that is very similar to that seen for EWM, with a peak incidence in young children and the elderly (Baker et al. 2012). Respiratory infections are intensely seasonal in temperate countries such as NZ. If their mortality burden dropped, in relative terms, from a range of measures (including improvements in living conditions, smaller household sizes, immunisation and better healthcare including the introduction of antibiotics and vaccines), then we would expect a corresponding drop in EWM. While in theory this hypothesis, or the role of changes in non-communicable disease rates in changes in EWM, could be tested by reviewing trends in cause of death, in practice such an analysis was not possible, for two reasons. First, understanding and classification of disease and causes of mortality has changed markedly over the time-period of our study, including in more recent decades (Link et al. 2011), making trend analysis unreliable. Second, even where disease understanding has been consistent, aggregations in available official NZ data have changed, preventing re-coding into usable data.

This last hypothesis would also have been supported if clear reductions in EWM had appeared following the introduction of influenza vaccination programmes, but no such change in trend appears. This absence of a structural break does not mean that influenza vaccination was ineffective, only that it is not possible to tell whether the on-going reductions in mortality rates and EWM since the 1990s were sustained due to vaccination, or whether the trend downwards would have continued regardless. Further investigation of this question is warranted, using post-1980 cause of death data.

### Study strengths and limitations

A clear strength of this study is its long 145-year time-period for a country with relatively robust mortality statistics. Nevertheless, while the mortality data were generally judged to be of good quality, they were not faultless. High mortality rates for males aged 20 to 29 in 1941 and 1942 in particular, and also 1943 to 1945, suggest data for that period likely include some war casualties, whether delayed deaths in NZ from injuries sustained overseas or deaths overseas being mis-recorded as occurring in NZ. Overseas war deaths may have been seasonal in line with ‘spring offensives’, but delayed deaths which occurred after return to NZ are unlikely to have been particularly seasonally responsive and will have flattened EWMIs in the early 1940s.

We considered removing sudden mass fatality events (List of disasters in New Zealand by death toll 2022; Wilson et al. 2017), predominantly from earthquakes, volcanic disasters, mining disasters and shipwrecks. However, doing so would have required age and sex data for the deceased to allow for standardisation, and these data were not widely available for mass fatality events early in the time period of interest. We therefore elected to leave these events in the data. While 1876 to 2020 mass fatality events were slightly more likely to occur in the June to September ‘winter’ months (37%; 26/71 mass death events), deaths from such events were less numerous in winter (20%; 529/2630 total deaths). Therefore, the inclusion of sudden mass fatality events will have reduced EWMIs in the relevant years rather than increased them.

While analysis of changes in the temperature-mortality relationship would have been preferable to the blunt EWMI measure, temperature data were not available for long enough, or for enough locations, to assign temperature exposure estimates to places of death across a wide timeframe. In addition, data for place of death were of variable quality, making further location-based analysis of questionable utility.

## Conclusions

NZ EWM increased significantly after the appearance of the 1918 influenza strain, but declined relatively steadily from the 1950s, with an average of 1450 excess winter deaths in the 2010s, about 4.5% of all deaths, compared to 7.9% of deaths in the 1920s. While population health measures and living standards have improved health year-round over the study period, the reduction in EWM is not clearly associated with any single societal intervention.

### Supplementary Information

Below is the link to the electronic supplementary material.

Supplementary file1 (PDF 173 KB)

## Data Availability

Statistics NZ population data and data subsequently extrapolated or interpolated by the authors for the current study are available from the corresponding author on reasonable request. NZ Department of Internal Affairs and NZ Ministry of Health data that support the findings of this study were used under license for the current study and so are not publicly available. Data may be made available by those government agencies or are available from the authors upon reasonable request and with permission of the government agency(s) concerned (NZ Department of Internal Affairs and/or NZ Ministry of Health).

## References

[CR1] Annual Report 18/19. (2019) Energy Efficiency and Conservation Authority. https://www.eeca.govt.nz/assets/EECA-Resources/Corporate-documents/EECA-Full-Annual-Report-2018-19.pdf. Accessed 26 July 2023

[CR2] Annual Report 2017/18. (2018) Energy Efficiency and Conservation Authority. https://www.eeca.govt.nz/assets/EECA-Resources/Corporate-documents/EECA-Annual-Report-2017-18.pdf. Accessed 26 July 2023

[CR3] Annual Report: 1 July 2019 - 30 June 2020. (2020) Te Tari Tiaki Pūngao: Energy Efficiency and Conservation Authority. https://www.eeca.govt.nz/assets/EECA-Resources/Corporate-documents/EECA-Annual-Report-2019-20.pdf. Accessed 25 July 2023

[CR4] Appendix 1: The history of immunisation in New Zealand. (2020) Ministry of Health. https://www.health.govt.nz/our-work/immunisation-handbook-2020/appendix-1-history-immunisation-new-zealand. Accessed 22 June 2022

[CR5] Baker MG, Telfar Barnard L, Kvalsvig A, Verrall A, Zhang J, Keall M, Wilson N, Wall T, Howden-Chapman P (2012) Increasing incidence of serious infectious diseases and inequalities in New Zealand: a national epidemiological study. Lancet 379(9821):1112–1119. 10.1016/S0140-6736(11)61780-7 10.1016/S0140-6736(11)61780-7 22353263

[CR6] Carson C, Hajat S, Armstrong B, Wilkinson P (2004) Changing patterns of winter- and temperature-related mortality in London in the twentieth century. Epidemiology 15(4):S93–S94. 10.1097/00001648-200407000-00233

[CR7] Carson C, Hajat S, Armstrong B, Wilkinson P (2006) Declining vulnerability to temperature-related mortality in London over the 20th century. Am J Epidemiol 164(1):77–84. 10.1093/aje/kwj147 10.1093/aje/kwj147 16624968

[CR8] Chau PH, Woo J (2015). The trends in excess mortality in winter vs. summer in a sub-tropical city and its association with extreme climate conditions. PloS One..

[CR9] Curwen M (1991). Excess winter mortality: a British phenomenon?. Health Trends.

[CR10] Davie GS, Baker MG, Hales S, Carlin JB (2007) Trends and determinants of excess winter mortality in New Zealand: 1980 to 2000. BMC Public Health 7:263. 10.1186/1471-2458-7-263 10.1186/1471-2458-7-263 PMC2174476 17892590

[CR11] de Schrijver E, Bundo M, Ragettli MS, Sera F, Gasparrini A, Franco OH, Vicedo-Cabrera AM (2022). Nationwide analysis of the heat- and cold-related mortality trends in Switzerland between 1969 and 2017: the role of population aging. Environ Health Perspect.

[CR12] Ditzen J, Karavias Y, Westerlund J (2021) Testing and estimating structural breaks in time series and panel data in Stata. https://arxiv.org/abs/2110.14550

[CR13] Ekamper P, van Poppel F, van Duin C, Garssen J (2009) 150 years of temperature-related excess mortality in the Netherlands. Demogr Res 21:385–425. https://www.demographic-research.org/volumes/vol21/14/21-14.pdf. Accessed 1 Nov 2023

[CR14] Excess winter mortality in England and Wales: 2019 to 2020 (provisional) and 2018 to 2019 (final) (2020). Office of National Statistics, UK. https://www.ons.gov.uk/peoplepopulationandcommunity/birthsdeathsandmarriages/deaths/bulletins/excesswintermortalityinenglandandwales/2019to2020provisionaland2018to2019final. Accessed 1 Nov 2023

[CR15] Fyfe C, Telfar L, Barnard, Howden-Chapman P, Douwes J (2020). Association between home insulation and hospital admission rates: retrospective cohort study using linked data from a national intervention programme. BMJ.

[CR16] Gasparrini A, Guo Y, Hashizume M, Lavigne E, Zanobetti A, Schwartz J, Tobias A, Tong S, Rocklov J, Forsberg B, Leone M, De Sario M, Bell ML, Guo YL, Wu CF, Kan H, Yi SM, De Sousa ZanottiStagliorio Coelho M, Saldiva PH, Honda Y, Kim H, Armstrong B (2015). Mortality risk attributable to high and low ambient temperature: a multicountry observational study. Lancet.

[CR17] Hajat S, Gasparrini A (2016) The excess winter deaths measure: why its use is misleading for publish health understanding of cold-related health impacts. Epidemiology 27(4):486–491. 10.1097/EDE.0000000000000479 10.1097/EDE.0000000000000479 PMC4890842 26986872

[CR18] Hales S, Blakely T, Foster RH, Baker MG, Howden-Chapman P (2012). Seasonal patterns of mortality in relation to social factors. J Epidemiol Community Health.

[CR19] Hanigan IC, Dear KBG, Woodward A (2021). Increased ratio of summer to winter deaths due to climate warming in Australia, 1968–2018. Aust N Z J Public Health.

[CR20] Hare EH, Moran PA, Macfarlane A (1981) The changing seasonality of infant deaths in England and Wales 1912–78 and its relation to seasonal temperature. J Epidemiol Community Health 35(2):77–82. 10.1136/jech.35.2.77 10.1136/jech.35.2.77 PMC1052128 7197709

[CR21] Hindley D (2020a) NZ vs Australia building regs. Build 180:84–85. https://www.buildmagazine.org.nz/assets/PDF/Build-180-84-Building-Controls-NZ-Vs-Australia-Building-Regs.pdf. Accessed 1 Nov 2023

[CR22] Hindley D (2020b) Rating our building regs - Part 1. Build 179:50–51. https://www.buildmagazine.org.nz/assets/PDF/Build-179-50-Feature-Building-Better-Rating-Our-Building-Regs-Part-1.pdf. Accessed 1 Nov 2023

[CR23] Hippocrates (400 B.C.E) On airs, waters and places. Available online at https://classics.mit.edu/Hippocrates/airwatpl.mb.txt. Accessed 1 Nov 2023

[CR24] Howden-Chapman P, Matheson A, Crane J, Viggers H, Cunningham M, Blakely T, Cunningham C, Woodward A, Saville-Smith K, O’Dea D, Kennedy M, Baker M, Waipara N, Chapman R, Davie G (2007) Effect of insulating existing houses on health inequality: cluster randomised study in the community. BMJ 334:460. 10.1136/bmj.39070.573032.80 10.1136/bmj.39070.573032.80 PMC1808149 17324975

[CR25] Huang QS, Wood T, Jelley L, Jennings T, Jefferies S, Daniells K, Nesdale A, Dowell T, Turner N, Campbell-Stokes P, Balm M, Dobinson HC, Grant CC, James S, Aminisani N, Ralston J, Gunn W, Bocacao J, Danielewicz J, Moncrieff T, McNeill A, Lopez L, Waite B, Kiedrzynski T, Schrader H, Gray R, Cook K, Currin D, Engelbrecht C, Tapurau W, Emmerton L, Martin M, Baker MG, Taylor S, Trenholme A, Wong C, Lawrence S, McArthur C, Stanley A, Roberts S, Rahnama F, Bennett J, Mansell C, Dilcher M, Werno A, Grant J, van der Linden A, Youngblood B, Thomas PG, Webby RJ (2021). Impact of the COVID-19 nonpharmaceutical interventions on influenza and other respiratory viral infections in New Zealand. Nat Commun.

[CR26] Immunisation Handbook 2017 (2018) 2nd edn. Ministry of Health, Wellington, New Zealand. https://researchspace.auckland.ac.nz/bitstream/handle/2292/47784/immshandbook-1-general-immunisation-principles-mar18-v2.pdf. Accessed 1 Nov 2023

[CR27] Indicators>Temperature (2020) Statistics NZ. https://www.stats.govt.nz/indicators/temperature. Accessed 9 May 2022

[CR28] Iparraguirre J (2014). Have winter fuel payments reduced excess winter mortality in England and Wales?. J Public Health.

[CR29] Jackson ML, Peterson D, Nelson JC, Greene SK, Jacobsen SJ, Belongia EA, Baxter R, Jackson LA (2014) Using winter 2009-2010 to assess the accuracy of methods which estimate influenza-related morbidity and mortality. Epidemiol Infect:1-9. 10.1017/s0950268814003276 10.1017/S0950268814003276 PMC9150941 25496703

[CR30] Jones RP, Ponomarenko A (2022) Trends in excess winter mortality (EWM) from 1900/01 to 2019/20-evidence for a complex system of multiple long-term trends. Int J Environ Res Public Health 19 (6). 10.3390/ijerph19063407 10.3390/ijerph19063407 PMC8953800 35329098

[CR31] Knutson V, Aleshin-Guendel S, Karlinsky A, Msemburi W, Wakefield J (2023). Estimating global and country-specific excess mortality during the COVID-19 pandemic. Ann Appl Stat.

[CR32] Ledesma JR, Isaac CR, Dowell SF, Blazes DL, Essix GV, Budeski K, Bell J, Nuzzo JB (2023). Evaluation of the Global Health Security Index as a predictor of COVID-19 excess mortality standardised for under-reporting and age structure. BMJ Global Health.

[CR33] Lerchl A (1998) Changes in the seasonality of mortality in Germany from 1946 to 1995: the role of temperature. Int J Biometeorol 42(2):84–88. 10.1007/s004840050089 10.1007/s004840050089 9923200

[CR34] Link J, Glazer C, Torres F, Chin K (2011). International Classification of Diseases coding changes lead to profound declines in reported idiopathic pulmonary arterial hypertension mortality and hospitalizations: implications for database studies. Chest.

[CR35] List of disasters in New Zealand by death toll. (2022) Wikipedia. https://en.wikipedia.org/wiki/List_of_disasters_in_New_Zealand_by_death_toll. Accessed 9 May 2022

[CR36] Maheswaran R, Chan D, Fryers PT, McManus C, McCabe H (2004) Socio-economic deprivation and excess winter mortality and emergency hospital admissions in the South Yorkshire Coalfields Health Action Zone, UK. Public Health 118:167–176. 10.1016/j.puhe.2003.09.004 10.1016/j.puhe.2003.09.004 15003406

[CR37] Marcuzzi G, Tasso M (1992). Seasonality of death in the period 1889–1988 in the Val di Scalve (Bergamo Pre-Alps, Lombardia, Italy). Hum Biol.

[CR38] McDowall M (1981). Long term trends in seasonal mortality. Population Trends.

[CR39] Mullan AB, Stuart SJ, Hadfield MG, Smith MJ (2010) Report on the Review of NIWA’s ‘Seven-Station’ Temperature Series. NIWA Information Series No. 78. NIWA, Wellington, New Zealand. https://niwa.co.nz/sites/niwa.co.nz/files/import/attachments/Report-on-the-Review-of-NIWAas-Seven-Station-Temperature-Series_v3.pdf. Accessed 1 Nov 2023

[CR40] nz.stat. (2021) Statistics NZ. https://www.stats.govt.nz/tools/nz-dot-stat. 2021. Accessed 1 Nov 2023

[CR41] Residential Tenancies (Smoke Alarms and Insulation) Regulations (2016). New Zealand. https://www.legislation.govt.nz/regulation/public/2016/0128/latest/DLM6856201.html. Accessed 25 September 2023

[CR42] Ross JC (1994) Some aspects of poliomyelitis in New Zealand. Post-Polio Support Society NZ Inc. https://www.moh.govt.nz/notebook/nbbooks.nsf/0/CD3582AA9BB60213CC256C07000C0D49/$file/some-aspects-of-poliomyelitis-NZ.pdf. Accessed 25 July 2023

[CR43] Singleton J (2008) An economic history of New Zealand in the nineteenth and twentieth centuries. In: EH.Net Encyclopedia, Whaples R (ed). https://eh.net/encyclopedia/an-economichistory-of-new-zealand-in-the-nineteenth-and-twentieth-centuries/. Accessed 1 Nov 2023

[CR44] Staddon PL, Montgomery HE, Depledge MH (2014). Climate warming will not decrease winter mortality. Nat Clim Change.

[CR45] Telfar Barnard L (2010) Home truths and cool admissions: New Zealand housing attributes and excess winter hospitalisation. University of Otago, Wellington. https://hdl.handle.net/10523/591. Accessed 1 Nov 2023

[CR46] The New Zealand official year-book, 1962 (1962) Department of Statistics, Wellington, New Zealand. https://www3.stats.govt.nz/New_Zealand_Official_Yearbooks/1962/NZOYB_1962.html. Accessed 1 Nov 2023

[CR47] Thermal insulation in New Zealand homes. (2020) Ministry for Culture and Heritage. https://nzhistory.govt.nz/page/thermal-insulation-required-nz-homes. Accessed 9 May 2020

[CR48] Tillett HE, Smith JW, Gooch CD (1983) Excess deaths attributable to influenza in England and Wales: age at death and certified cause. Int J Epidemiol 12(3):344–352. 10.1093/ije/12.3.344 10.1093/ije/12.3.344 6629624

[CR49] White V, Jones M (2017) Warm, dry, healthy? Insights from the 2015 House Condition Survey on insulation, ventilation, heating and mould in New Zealand houses. BRANZ, Porirua, NZ. https://www.branz.co.nz/documents/857/SR372_Warm_dry_healthy.pdf. Accessed 1 Nov 2023

[CR50] Wilkinson P, Pattenden S, Armstrong B, Fletcher A, Kovats RS, Mangtani P, McMichael AJ (2004) Vulnerability to winter mortality in elderly people in Britain: population based study. BMJ 329(7467):647–651. 10.1136/bmj.38167.589907.55 10.1136/bmj.38167.589907.55 PMC517639 15315961

[CR51] Wilson N, Barnard LT, Summers JA, Shanks GD, Baker MG (2012). Differential mortality rates by ethnicity in 3 influenza pandemics over a century, New Zealand. Emerg Infect Dis.

[CR52] Wilson N, Morales A, Guy N, Thomson G (2017). Marked decline of sudden mass fatality events in New Zealand 1900 to 2015: the basic epidemiology. Aust N Z J Public Health.

[CR53] Woodward A, Blakely T (2015). The healthy country. A history of life & death in New Zealand.

