# Supplementary material for: The rise and fall of excess winter mortality in New Zealand from 1876 to 2020
Source: Int J Biometeorol. 2023 Nov 27;68(1):89–100. doi: 10.1007/s00484-023-02573-6 (PMC10752914; doi:10.1007/s00484-023-02573-6)
Supplement: Supplementary file 1 — Supplementary file1 (PDF 173 KB) [file 484_2023_2573_MOESM1_ESM.pdf]

Online Resource 1. Statistical details for structural break points.

(a) All ages, EWM: No additional breaks found for 12 breaks.

Sequential test for multiple breaks at unknown breakpoints, Bai & Perron Critical Values:

	Test statistic	1% Critical value	5% Critical value	10% Critical value
F(1|0)	62.49	13.58	9.63	8.02
F(2|1)	3.56	15.03	11.14	9.56
F(3|2)	5.55	15.62	12.16	10.45
F(4|3)	5.87	16.39	12.83	11.07
F(5|4)	2.56	16.6	13.45	11.65
F(6|5)	1.29	16.9	14.05	12.07
F(7|6)	2.84	17.04	14.29	12.47
F(8|7)	5.33	17.27	14.5	12.7
F(9|8)	0.67	17.32	14.69	13.07
F(10|9)	1.88	17.61	14.88	13.34
F(11|10)	1.61	17.61	14.88	13.34
Detected number of breaks		1	1	1

Maximum number of breaks reached with null always rejected.

Estimation of break points: T = 135, SSR = 0.53, Trimming = 0.05

#	Index	Date	95% Conf. Interval
1	33	1918	-2.7e+04 - 3.1e+04

(b) All ages, winter mortality rates: No additional breaks found for 14 breaks.

Sequential test for multiple breaks at unknown breakpoints, Bai & Perron Critical Values:

	Test statistic	1% Critical value	5% Critical value	10% Critical value
F(1|0)	77.08	13.58	9.63	8.02
F(2|1)	5.21	15.03	11.14	9.56
F(3|2)	20.65	15.62	12.16	10.45
F(4|3)	18.46	16.39	12.83	11.07
F(5|4)	7.4	16.6	13.45	11.65
F(6|5)	5.44	16.9	14.05	12.07
F(7|6)	3.71	17.04	14.29	12.47
F(8|7)	6.49	17.27	14.5	12.7
F(9|8)	1.69	17.32	14.69	13.07
F(10|9)	0.54	17.61	14.88	13.34
F(11|10)	0.85	17.61	14.88	13.34
F(12|11)	1.39	17.61	14.88	13.34
F(13|12)	1.2	17.61	14.88	13.34
Detected number of breaks	(min)	1	1	1
	(max)	4	4	4

Estimation of break points: T=134, SSR = 219.28, Trimming=0.05

#	Index	Date	95% Conf. Interval
1	33	1918	1895 - 1941

(c) All ages, non-winter mortality rates: No additional breaks found for 14 breaks.

Sequential test for multiple breaks at unknown breakpoints, Bai & Perron Critical Values:

	Test statistic	1% Critical value	5% Critical value	10% Critical value
F(1|0)	62	13.58	9.63	8.02
F(2|1)	11.14	15.03	11.14	9.56
F(3|2)	19.26	15.62	12.16	10.45
F(4|3)	21.07	16.39	12.83	11.07
F(5|4)	4.31	16.6	13.45	11.65
F(6|5)	2.72	16.9	14.05	12.07
F(7|6)	9.32	17.04	14.29	12.47
F(8|7)	1.09	17.27	14.5	12.7
F(9|8)	1.34	17.32	14.69	13.07
F(10|9)	5.89	17.61	14.88	13.34
F(11|10)	1.48	17.61	14.88	13.34
F(12|11)	0.1	17.61	14.88	13.34
F(13|12)	0.26	17.61	14.88	13.34
Detected number of breaks	(min)	1	4	4
	(max)	4	4	4

Estimation of break points: T=134, SSR = 53.97, Trimming=0.05

#	Index	Date	95% Conf. Interval
1	53	1938	1824 - 2052
2	60	1945	1818 - 2072
3	80	1965	-3.1e+03 - 7006
4	103	1988	1715 - 2261

(d) 65 years and over, EWMI: No additional breaks found for 14 breaks.

Sequential test for multiple breaks at unknown breakpoints, Bai & Perron Critical Values:

	Test statistic	1% Critical value	5% Critical value	10% Critical value
F(1|0)	39.46	13.58	9.63	8.02
F(2|1)	7.99	15.03	11.14	9.56
F(3|2)	11.03	15.62	12.16	10.45
F(4|3)	2.2	16.39	12.83	11.07
F(5|4)	2.87	16.6	13.45	11.65
F(6|5)	12.8	16.9	14.05	12.07
F(7|6)	1.8	17.04	14.29	12.47
F(8|7)	1.91	17.27	14.5	12.7
F(9|8)	2.23	17.32	14.69	13.07
F(10|9)	1.99	17.61	14.88	13.34
F(11|10)	2.32	17.61	14.88	13.34
Detected number of breaks	(min)	1	1	1
	(max)	1	1	6

Null hypothesis rejected more than once after non-rejection. The detected number of breaks indicates the minimum and maximum number of breaks for which the null hypothesis is rejected.

Estimation of break points: T=135, SSR = 0.85, Trimming=0.05

#	Index	Date	95% Conf. Interval
1	33	1918	-2.9e+04 - 3.3e+04

(e) 65 years and over, winter mortality rates: No additional breaks found for 17 breaks.

Sequential test for multiple breaks at unknown breakpoints, Bai & Perron Critical Values:

	Test statistic	1% Critical value	5% Critical value	10% Critical value
F(1|0)	77.27	13.58	9.63	8.02
F(2|1)	64.2	15.03	11.14	9.56
F(3|2)	16.73	15.62	12.16	10.45
F(4|3)	14.14	16.39	12.83	11.07
F(5|4)	7.71	16.6	13.45	11.65
F(6|5)	8.14	16.9	14.05	12.07
F(7|6)	5.24	17.04	14.29	12.47
F(8|7)	4.06	17.27	14.5	12.7
F(9|8)	4.71	17.32	14.69	13.07
F(10|9)	1.07	17.61	14.88	13.34
F(11|10)	0.29	17.61	14.88	13.34
F(12|11)	0	17.61	14.88	13.34
F(13|12)	0.04	17.61	14.88	13.34
F(14|13)	0.11	17.61	14.88	13.34
F(15|14)	1.88	17.61	14.88	13.34
F(16|15)	0.02	17.61	14.88	13.34
Detected number of breaks		3	4	4

The detected number of breaks indicates the highest number of breaks for which the null hypothesis is rejected.

Estimation of break points: T=135, SSR = 5376.95, Trimming=0.05

#	Index	Date	95% Conf. Interval
1	15	1900	1895 - 1905
2	32	1917	1916 - 1918
3	75	1960	1874 - 2046
4	104	1989	1974 - 2004

(f) 65 years and over, non-winter mortality rates: No additional breaks found for 13 breaks.

Sequential test for multiple breaks at unknown breakpoints, Bai & Perron Critical Values:

	Test statistic	1% Critical value	5% Critical value	10% Critical value
F(1|0)	93.29	13.58	9.63	8.02
F(2|1)	25.07	15.03	11.14	9.56
F(3|2)	18.49	15.62	12.16	10.45
F(4|3)	22.19	16.39	12.83	11.07
F(5|4)	11.34	16.6	13.45	11.65
F(6|5)	17.66	16.9	14.05	12.07
F(7|6)	2.62	17.04	14.29	12.47
F(8|7)	2.67	17.27	14.5	12.7
F(9|8)	3.54	17.32	14.69	13.07
F(10|9)	1.04	17.61	14.88	13.34
F(11|10)	2.32	17.61	14.88	13.34
F(12|11)	1.27	17.61	14.88	13.34
Detected number of breaks	(min)	4	4	4
	(max)	6	6	6

Null hypothesis rejected more than once after non-rejection. The detected number of breaks indicates the minimum and maximum number of breaks for which the null hypothesis is rejected.

Estimation of break points: T=135, SSR = 2271.05, Trimming=0.05

#	Index	Date	95% Conf. Interval
1	11	1896	1888 - 1904
2	28	1913	1881 - 1945
3	48	1933	1881 - 1985
4	104	1989	1968 - 2010
